# An Unexpected Cause of Bradycardia in a Patient with Bacterial Meningitis

**DOI:** 10.1155/2017/4297372

**Published:** 2017-06-21

**Authors:** Petros Ioannou, Magdalini Velegraki, Stella Soundoulounaki, Achilleas Gikas, Diamantis P. Kofteridis

**Affiliations:** Department of Internal Medicine, University Hospital of Heraklion, Crete, Greece

## Abstract

Sinus bradycardia which is a sinus rhythm with a resting heart rate of less than 60 bpm is caused by intrinsic cardiac disorders like sick sinus syndrome or inferior myocardial infarction, metabolic and environmental causes (such as hypothyroidism and electrolyte disorders), medications (such as beta-blockers and amiodarone), infection (such as myocarditis), increased intracranial pressure, and toxic exposure, while it can sometimes be a normal phenomenon, especially during sleep, in athletes, and during pregnancy. Symptomatic sinus bradycardia should warrant a thorough work-up in order to identify any reversible causes; otherwise, placement of a permanent pacemaker could be needed. We present the case of a patient who was admitted due to confusion and fever and was found to have pneumococcal meningitis and bacteremia, and during his hospital stay he developed symptomatic sinus bradycardia that was of intractable cause and persistent. Placement of a permanent pacemaker was chosen until the night staff of the hospital discovered by chance the neglected cause of his bradycardia.

## 1. Introduction

Sinus bradycardia which is a sinus rhythm with a resting heart rate of less than 60 bpm is rarely symptomatic until the heart rate is less than 50 bpm and sometimes can be a normal phenomenon, especially during sleep, in well-conditioned athletes, and during pregnancy and in some elderly patients [1, 2]. Some other causes of sinus bradycardia can be associated with intrinsic cardiac disorders like sick sinus syndrome or inferior myocardial infarction, metabolic and environmental causes (such as hypothermia, hypothyroidism, hypoglycemia, and electrolyte disorders), medications (such as digitalis glycosides, beta-blockers, calcium channel-blockers, and amiodarone), infection (such as myocarditis, diphtheria, or rheumatic fever), increased intracranial pressure, and toxic exposure to toluene, dimethyl sulfoxide (DMSO), or other toxins [1–4]. Development of symptomatic sinus bradycardia should warrant treatment in order to reduce symptoms as well as a thorough work-up in order to identify any reversible causes of bradycardia and prevent the unnecessary placement of a permanent pacemaker that could be otherwise needed in the case of an intractable and persistent sinus bradycardia.

We present the case of a patient hospitalized due to bacterial meningitis that developed intractable symptomatic sinus bradycardia during his hospitalization and was chosen to undergo a permanent pacemaker placement until an observation by the night staff of the hospital was made.

## 2. Case Report

A 70-year-old male was admitted to our hospital because of a severe headache, fever, and confusion. The patient had been well until 5 hours before admission, when fever up to 39.6°C and mental status change developed. He had a history of Waldenstrom macroglobulinemia last treated 10 months ago, chronic HBV infection, chronic obstructive pulmonary disease, hypertension, and benign prostate hyperplasia.

On examination, he was confused, with a Glasgow Coma Scale of 11/15 (eye opening response 2/4, best verbal response 4/5, and best motor response 5/6), and febrile and had nuchal rigidity. Due to presumed bacterial meningitis, he was immediately treated with ceftriaxone, vancomycin, ampicillin, and dexamethasone, according to European guidelines on empiric treatment of bacterial meningitis, while a computed tomography (CT) of the head was performed due to decreased level of consciousness and did not reveal any abnormality. A lumbar puncture was performed and revealed 6960 white blood cells (WBCs) per field at the cerebrospinal fluid (CSF), while 98% were neutrophils. Latex and gram stain of the CSF were negative, while a culture of the CSF and blood cultures were sent. The patient was admitted to the Internal Medicine Department. Ceftriaxone, vancomycin, ampicillin, and dexamethasone were continued. On the fourth day of hospitalization, both CSF and blood cultures grew* S. pneumoniae* that was sensitive to penicillin and then antimicrobial treatment was deescalated to ceftriaxone only. On the third day of hospitalization, his fever and symptoms resolved completely, while the inflammatory markers decreased.

**Figure 1:**
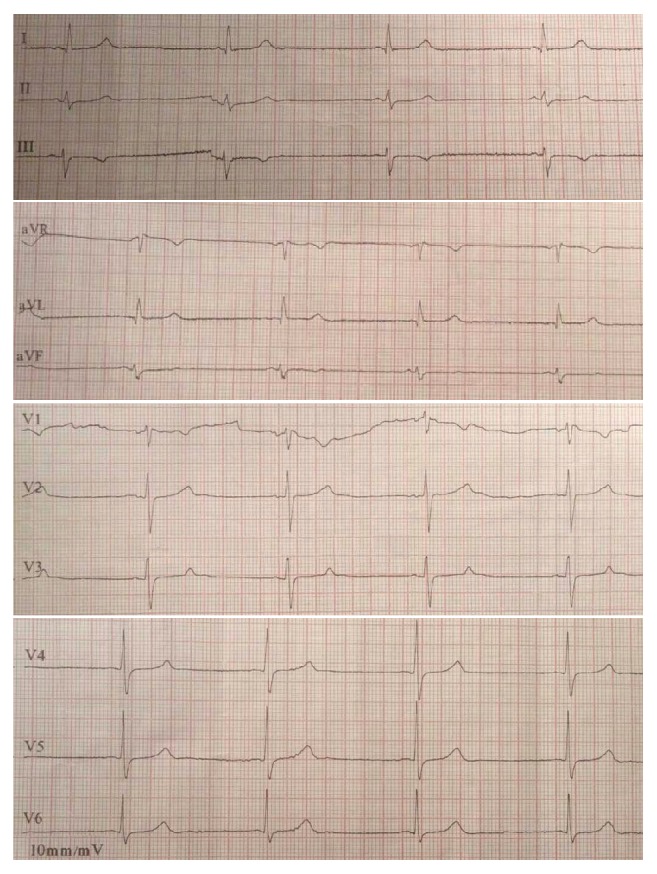
Electrocardiogram of the patient taken on the fourth day of his hospitalization due to dizziness and a low heart rate, showing sinus bradycardia.

However, on the fourth day of his hospitalization, the patient developed bradycardia down to 40 beats per minute, which was associated with mild dizziness while standing without improvement of the heart rate, with the electrocardiogram showing a sinus bradycardia (Figure 1). Because of the CNS infection, the possibility of intracranial hypertension was suspected, and a fundoscopy was performed, which did not show signs of increased intracranial pressure. Since no other cause of bradycardia was noted, even after all medications were recalled, a 24-hour rhythm recording (Holter) was performed, which revealed sinus bradycardia as a basal rhythm during the 24 hours, with a lowest heart rate of 30 beats per minute, as well as three episodes of nonsustained ventricular tachycardia. A transthoracic heart ultrasound was performed and revealed an ejection fraction of 55%, mild left ventricular hypertrophy, left atrial dilation, and a small amount of pericardial fluid. Due to the absence of an obvious reversible factor that could be associated with the bradycardia and the presence of symptoms, the placement of a permanent pacemaker was considered. Surprisingly, some days before the placement of the pacemaker, a medical doctor that was on a night shift discovered that the patient's wife was administering to him eye drops containing timolol, a b-blocker used for glaucoma, which she started doing the night before the bradycardia had developed, without letting any member of the staff know. The eye drops were stopped and the bradycardia resolved along with the patient's symptoms. A repeat 24-hour rhythm recording (Holter) was performed the following days and was normal. The patient was discharged and during follow-up there was no recurrence of the bradycardia.

## 3. Discussion

Sinus bradycardia (sinus rhythm of less than 60 bpm) can be normal in specific instances, but specific causes such as ischemic heart disease, sick sinus syndrome, and noncardiac causes such as metabolic causes, medications, and toxins exist [1–4]. Bacterial meningitis is an inflammatory disease of the meninges caused by bacteria, manifesting with fever, change in mental status, headache, nuchal rigidity, seizures, increased intracranial pressure, and focal neurologic findings, caused mainly by* S. pneumoniae*,* N. meningitidis*, and* L. monocytogenes* [5, 6]. In our case, the patient presented acutely with bacterial meningitis that was treated promptly with antibiotics according to European guidelines for bacterial meningitis [7] and responded rapidly, but even though the following days the symptoms associated with the CNS infection resolved, surprisingly, sinus bradycardia developed, which was also associated with dizziness. The only obvious cause in this case would be either intracranial hypertension or medication. Intracranial hypertension was unlikely given the normal CT at admission, the normal neurologic examination the days after initiation of the antibiotics, the improvement of the symptoms associated with the CNS infection, the reduction of the inflammatory parameters, and the sterilization of the blood cultures; however, a fundoscopy was performed, which did not show signs of increased intracranial pressure. On the other hand, the medication administered could not be associated with bradycardia, with the exception of glucocorticoids, which have been implicated in bradycardia in case reports but in quite higher doses than those administered in our case [8, 9]. Since the patient was symptomatic and an obvious cause of bradycardia could not be identified after a work-up with a 24-hour rhythm recording (Holter) and a transthoracic heart ultrasound, placement of a permanent pacemaker was considered. In our surprise, even though the patient's medications were revised daily in order to identify any missing medication that could be causing the bradycardia, the patient's wife was (accidentally) found to be giving to the patient beta-blocker (timolol) containing eye drops for glaucoma, which are known to be causing bradycardia [10]. Interestingly, no other cause of bradycardia has been found, implying that a missing medication was the reason for the patient's bradycardia.

This case report reveals a neglected cause of sinus bradycardia, suggesting that awareness is needed for “hidden” medications, like beta-blocker containing eye drops, in order to avoid unnecessary interventions, like placing a permanent pacemaker for a drug induced symptomatic sinus bradycardia.
